# A Narrative Review of the Advances in Screening Methods for Diabetic Retinopathy: Enhancing Early Detection and Vision Preservation

**DOI:** 10.7759/cureus.53586

**Published:** 2024-02-04

**Authors:** Tanisha Upadhyay, Roshan Prasad, Swapneel Mathurkar

**Affiliations:** 1 Ophthalmology, Jawaharlal Nehru Medical College, Datta Meghe Institute of Higher Education and Research, Wardha, IND; 2 Medicine, Jawaharlal Nehru Medical College, Datta Meghe Institute of Higher Education and Research, Wardha, IND

**Keywords:** vision protection, early detection, biomarkers, screening methods, prevalence, diabetes retinopathy

## Abstract

Diabetes mellitus (DM) is putting a great burden worldwide. This rise in DM cases, both type 1 and 2, significantly impacts public health. India has grappled with a diabetes epidemic for several years, leading to many misdiagnosed and untreated diabetes cases. Diabetes remains a significant factor in adult-onset blindness despite improvements in diabetes management. This increases the danger of diabetic retinopathy (DR) with permanent loss of sight for those affected. The screening for DR aims to identify those persons with complications arising from diabetes or DR, which could potentially result in blindness, so that treatment can be started immediately and blindness can be avoided. A comprehensive health system approach is required to ensure that the public sector in India effectively screens for DR. Improving patient outcomes and avoiding visual loss depends significantly on early identification and treatment.

This article discusses the actions that should be implemented to establish a national effort for systematic DR screening. It also highlights the importance of screening in DR and its impact on treatment effectiveness. Regular screenings enable the early detection of retinopathy, allowing for timely intervention and treatment. Early screening helps prevent complications associated with DR, such as macular edema or retinal detachment. Screening also assists healthcare providers in planning, optimizing treatment approaches, and monitoring treatment effectiveness. Meanwhile, early intervention is essential for enhancing treatment outcomes, thus enhancing the chances of preserving vision and preventing further progression of the disease. This helps in improving the overall management of this sight-threatening complication.

## Introduction and background

Diabetes mellitus (DM), which is among the major health challenges of the present day, has a substantial effect on both socioeconomic growth and public health globally. In most other industrialized and emerging nations, the incidence and prevalence of DM has increased in recent decades [1]. As of 2017, 451 million (m) adults worldwide were predicted by the International Diabetes Federation (IDF) to have diabetes. Till 2,045 years, this number is projected to jump to more than 690m if no effective preventive measures are employed. Adolescents and children under 20 years of age are projected to have more than a million cases of type 1 DM, and the prevalence of both type 1 and 2 DM (T2DM) has also increased in this age group [1]. However, type 1 diabetes is not the only condition that is rising in prevalence among youth. A projected 22m children globally are overweight or obese, and it has been established that there is a rise in type 2 diabetes with an early onset among young people who are obese [2].

Diabetes is highly linked with vascular alterations, including structural, anatomical, and functional changes, which can lead to tissue and multi-organ dysfunction. These include both macro- and microvascular complications. Peripheral vascular disease, ischemic heart disease, and cerebrovascular diseases are examples of macrovascular complications, whereas neuropathy, retinopathy, and nephropathy are examples of microvascular complications [3]. Perhaps the most rampant microvascular consequence of diabetes is diabetic retinopathy (DR). In the United States alone, it is to blame for about 10,000 new cases of blindness yearly. The duration and severity of hyperglycemia influence the threat for the development of DR and other microvascular consequences of DM. It has also been discovered that the development of DR in individuals with T2DM is correlated to the severity of hyperglycemia along with the presence of hypertension [3].

DR is a serious microvascular consequence of DM, which can cause blindness. The elevated blood sugar levels in diabetes harm retinal blood vessels. These vascular structures ultimately dilate and burst [4]. When these microscopic blood vessels leak blood and other fluids, it results in DR. This leads to edema in the tissue of the retina, leading to its swelling and obscuring or clouding of vision. In working-age individuals, DR causes significant worsening of vision, due to the growth of aberrant new blood vessels on the retina. Thus, DR must be diagnosed by locating these abnormal blood vessel changes in the retina. Nonproliferative DR (NPDR) and proliferative DR (PDR) are the two clinical phases of DR [4].

The initial stages of NPDR show the development of retinal abnormalities such as microaneurysms, hemorrhages, and hard exudates owing to the increased permeability and obstruction in the retinal blood vessels. Even if the patients don't have any symptoms, fundus photography can find these abnormalities [5]. The development of fresh blood vessels on the retina is called neovascularization, a more severe stage of PDR. If these aberrant new blood vessels leak into the vitreous, causing vitreous hemorrhage or if there is tractional retinal detachment, patients can complain of severe vision loss at this time [6]. Diabetic macular edema (DME) is the primary reason for vision loss in patients of DR [6]. One defining symptom of DME is macular swelling or thickening, which is instigated by the collection of fluid in macula both inside and beneath retina. This fluid accumulates due to the blood-retinal barrier (BRB) collapse. DM can induce DME, which impairs vision and reduces visual acuity at any stage of DR [7].

This review aims to delve into the intricate landscape of DR, thoroughly examining its etiology, prevalence, clinical pathophysiology, molecular pathology, screening modalities, challenges and significance of early diagnosis, and intervention. The primary objectives of this review are to synthesize existing knowledge, highlight recent advancements in the screening modalities of DR, and provide clinicians and researchers with a comprehensive resource for understanding and navigating the complexities of prolonged diabetes, which leads to DR, enhancing early detection and intervention of DR, thus contributing to vision preservation. By addressing these objectives, we strive to contribute to the ongoing discourse on DR, fostering informed decision-making in clinical practice and guiding future avenues of research.

## Review

Methodology

A thorough search of the internet databases, including PubMed and Scopus, was done to conduct the review. Relevant articles were searched using the keywords "diabetes retinopathy," "prevalence," "screening techniques," "indicators," "early identification," and "vision preservation." The inclusion criteria included articles published in peer-reviewed publications, written in English, and focusing on DR, its prevalence in the community, and screening techniques for its early detection and vision protection. Articles published between the years 2013 and 2023 were included in our study to ensure the inclusion of recently published articles, which provide value for comprehensive research. Manual searches of reference lists were also carried out to find other pertinent studies. Original research papers as well as review papers were taken into consideration for inclusion. The papers that focused on the prevalence, causes, significant discoveries in screening methods, challenges in screening, and strategies for early eyesight restoration were included in our study. The rest of them were excluded. Our exclusion criteria included duplicate articles, articles published in non-peer-reviewed journals or written in a language other than English, and those with missing or inaccessible full texts. We ensured that only those research papers that fit the preset criteria were included. Titles, abstracts, and full-text papers were screened during selection. The authors reached a consensus to settle disagreements on the choice of studies.

Prevalence and risk factors of DR

The rising prevalence of DM in low- and middle-income (LMI) countries may be accredited to a number of factors, including urbanization, sedentary lifestyles, dietary changes that include more refined foods, a lack of treatment facilities, and treatment delays. Between 2010 and 2030, the percentage of grown-ups with diabetes is predicted to rise nearly by 70% in low- and middle-income nations, while it will only rise by 20% in high-income nations. By 2045, the number of diabetics is predicted to increase by 500% in many Southeast Asian (SEA) nations [8].

A previous study by Lee et al. indicated that the incidence and prevalence of DR are greater in high-income western nations as compared to that in LMI Asian nations; the USA, Canada, the UK, and Australia had rates between 29% and 40%, whereas South Korea had 15.8%, India had 18%, and China had 23% [9]. Recent research and a meta-analysis, however, indicated that the frequency of DR may be higher than anticipated in Asian nations. The lower rates might have resulted from inadequate access to healthcare, delayed diagnosis of DR, and a lack of awareness [10].

Worldwide, DR's incidence and sickness impact are anticipated to rise significantly over the following years, increasing from roughly 103m persons in 2020 to 131m in 2030 and 160m by 2045 [11]. These predictions result from several causes, such as the rising incidence of diabetes worldwide, alterations in lifestyle, longer life expectancies, and an aging world population. The abrupt increase in DR illness prevalence of over 25% in a decade is expected to put further added stress on the current overburdened healthcare resources and systems [12].

Even though better DM management has led to a recent decrease in incidence and prevalence of all stages of DR globally, the crude prevalence of blindness and visual impairment brought on by DR augmented globally between 1991 and 2016, mainly as a result of the rising incidence of T2DM, especially in LMI countries. The rising incidence and prevalence of DM in LMI countries can be attributed to several factors, including urbanization, sedentary lifestyles, and dietary changes that include more refined foods leading to obesity. These lead to the predevelopment of T2DM and obesity-induced T2DM. This increased population at risk for T2DM is ultimately at increased risk for developing DR. Early DR detection and fast treatment can help diabetic people keep their normal vision [8,9].

Major Risk Factors for DR

Table 1 shows the major risk factors for DR [13].

**Table 1:** Risk factors for diabetic retinopathy Adopted from article [13].

Sl. No.	Risk Factor	Effect on Diabetic Retinopathy (DR)
1.	Diabetes mellitus (HbA1c)	Every 1% decrease in HbA1c levels equals a decrease in 40% of DR, 25% decreased need for retinal photocoagulation, and a decrease in 15% cases of blindness due to DR.
2.	Systolic blood pressure (SBP)	Every 10 mmHg decrease in SBP equals a reduction in 35% cases of DR, 35% decreased need for retinal photocoagulation, and a decrease in 50% cases of blindness due to DR.
3.	Hyperlipidemia	Increased triglyceride levels are associated with the causation of DR, while increased low-density cholesterol (LDL) or decreased high-density lipoprotein (HDL) levels, as well as a high HDL/LDL ratio, are associated with the causation of DME.
4	Body mass index (BMI)	A BMI of >31 kg in males and a BMI of >32 kg in females and a BMI of <20kg in both genders were associated with increased risk for the causation of DR.

Another study by Yin et al. showed that among patients with DM, DR was the most prevalent ocular fundus illness. In addition, there are several independent risk factors for DR, such as age (P = .0003), male sex (P = .018), hypertension (P < .0001), long-term diabetes (P < .0001), fasting blood glucose (P < .0001), serum total cholesterol (P < .0001), serum triglyceride (P = .0006), high body mass index(BMI) (P < .0001) and %HbA1c (P < .0001) [14].

Pathophysiology of DR

Disruption of the BRB is a common symptom of DR. This failure results in blood components seeping into the retinal tissue, which frequently manifests in DM, mainly due to the degradation of the inner BRB. Vascular endothelial growth factor (VEGF) is potent in increasing vascular permeability and is closely linked to blood vessel leakage [15]. Additionally, DR frequently involves thickening of the vascular basement membrane. The overexpression of fibronectin, collagen, and laminin is thought to be responsible for this thickening [16].

**Figure 1:**
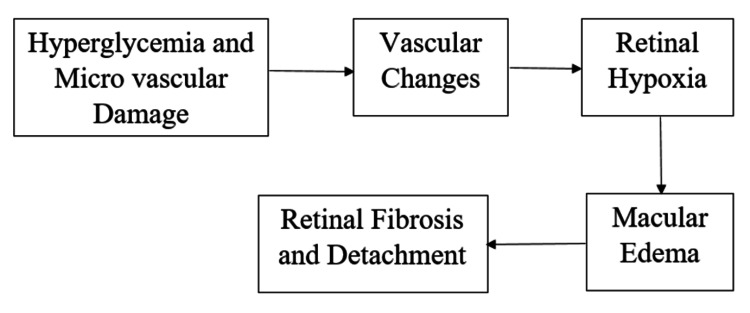
Events involved in the pathogenesis of diabetic retinopathy Developed from the article [17].

The malfunction and loss of pericytes are essential components of the pathological alterations in capillaries seen in DR. Pericytes preserve proper retinal function by promoting vascular endothelial cells' differentiation, movement, and growth. As a result, "pericyte ghost" and the disappearance of pericytes are important histological indications of DR [17]. Figure 1 shows events involved in the pathogenesis of DR.

Molecular pathology of DR

Stimulation of Protein Kinase-C (PKC)

The disease's underlying causes are still poorly known in many cases due to the biology of DR, which is incredibly complicated. Protein kinase-C is an intracellular signaling protein that has attracted much attention as it plays a crucial role in developing many ocular complications. Increased glucose flow is also a result of hyperglycemia via the glycolysis route. As a result, diacylglycerol (DAG) production is increased, activating the PKC pathway [18]. The induced production of stress-associated proteins, like the heat shock protein and C-Jun kinases, two essential mediators of vascular function, through the stimulation of mitogen-activated protein kinase (MAPK) factor, is one of the molecular processes impacted when PKC is overexpressed in DR [19]. In particular, it has been demonstrated that the PKC isoform promotes the synthesis of VEGF, a crucial regulator of angiogenesis, and vascular permeability that underlies the molecular pathophysiology of DR. The increased activity of nicotinamide-adenine dinucleotide phosphate (NADPH) oxidase with nuclear factor kappa B (NF-KB) in many cells, which includes pericytes, smooth muscle cells, and endothelial cells, is also promoted by PKC activation, which exacerbates DR's oxidative stressors and inflammatory pathophysiological components [20].

A number of PKC inhibitors have been studied. Ruboxistaurin is an oral PKCβ inhibitor that has shown promising results in treating DR and DME by reducing cellular and peripheral blood flow disruptions and reducing the associated cellular and vascular changes, as found in in-vitro as well as in-vivo studies. Control trials have been carried out during the past 10 years, and both preclinical and clinical investigations have shown the positive effect of ruboxistaurin. The PKC phase-II trial has shown that ruboxistaurin can lessen visual loss in people with DR, and DME appears to respond both anatomically and functionally to ruboxistaurin. The FDA has given the producer of drug ruboxistaurin permission to use it to prevent loss of vision in patients of DR. However, until more trials for this indication are completed, the drug is not yet available for clinical use [19,21].

Other biochemical pathways for the pathogenesis of DR

The Polyol Route

Inadequate DR medication-induced hyperglycemia results in an aberrant metabolic pathway activity, which triggers aldose reductase to convert the surplus glucose to sorbitol. This sorbitol accumulates inside the cells since it cannot cross the cellular membranes, leading to osmotic damage, among other problems. When sorbitol is converted into fructose as deoxyglucose and fructose-3 phosphates using the enzyme sorbitol dehydrogenase, both of which are powerful glycolysis-causing agents, advanced glycation end products (AGEs) are created [7].

Additionally, the activation of the polyol pathway reduces NADPH availability, making the affected cells more vulnerable to oxidative damage. As a result of this, the reduced glutathione pool is depleted, increasing the levels of ROS. Recent research also suggests that downregulating the nicotinamide adenine dinucleotide (NAD+)-depleting NADH oxidase and suppressing NADH-dependent dehydrogenase enzyme activity may worsen the ROS-induced shift in NADH/NAD+ levels caused by sorbitol dehydrogenase. Decreased GAPDH activity has been linked to DR as a causal metabolic route, as will be explained later [21].

Synthesis of AGEs

As a result of having access to glucose more easily, diabetes patients have noticeably more significant levels of AGE generation and accumulation. The ability of AGEs to bond together with amino acids, so changing both physical and chemical makeup, is one of their primary pathogenic traits. This comprises blood vessel wall components, cellular receptors, and basement membranes in diabetes. Additionally, AGEs can stimulate the receptor linked to pro-inflammatory and prooxidant activities, increasing leukocyte adherence and oxidative stress in DR patients [22].

The molecular pathophysiology of DR has been linked to these pathways recently. In diabetic rats given aminoguanidine hydrochloride, an AGE generation inhibitor, pericyte loss has been associated with developing acellular capillaries and accumulating AGEs. Similarly, treatment with the AGE production inhibitor pyridoxamine, a vitamin B6 derivative, lowered the expression of molecules in the basement membrane and avoided capillary washout [20].

Poly (ADP-Ribose) Polymerase (PARP) Activation

It has been established that increased levels of ROS-mediated DNA damage-dependent PARP stimulation are linked to hyperglycemia-induced oxidative stress. Consequently, NAD+ is depleted and GAPDH is concurrently inhibited as a result of the enzyme's catalytic cofactor being lost and PARP-mediated ribosylation [23]. Together, these molecular processes have been demonstrated to be involved in the malfunctioning of endothelial cells in diabetic blood arteries, including DR. Additionally, inhibiting PARP was shown to protect against diabetes-related retinopathy, supporting its involvement in DR pathogenesis [24].

Activation of the Renin-Angiotensin-Aldosterone System (RAAS)

The endocrine mechanism that controls systemic blood pressure is known as the RAAS. Although the precise role of the RAAS within DR is still unknown, evidence suggests that it is upregulated in DR patients, and its function as a regulator of vascular hydrodynamics implicates it in the development and progression of the disease [25]. Local glucose and its succinate metabolite are accumulated before the RAAS in DR is activated. In turn, this causes the G-protein-coupled receptor GPR91 to become active, prompting the release of prorenin and renin from juxtaglomerular cells. Furthermore, capillary perfusion and vascular structure have been observed to be negatively impacted by the angiotensin-converting enzyme (ACE) expression in the retina. Here, it has been demonstrated and linked to accelerated DR progression that ACE-mediated VEGF overexpression exists. Clinical research on humans has supported these findings, showing that lisinopril-mediated ACE inhibition can stop the growth of new blood vessels in diabetic eyes. Another study that supports this finding found that diabetic participants' neovascularization was inhibited by losartan-mediated suppression of the angiotensin-II type I receptor [26].

Prevalence of DR in India and the role of screening

According to the IDF, India had over 77m individuals with diabetes in 2019 and is anticipated to have 101m by 2030, with a major portion of undiagnosed diabetes [26]. More than half of those with diabetes go untreated, which is a concerning statistic given that complications are typically not discovered until after the disease has progressed. The fact that more than a third of people with diabetes have never had their eyes examined is even more concerning. According to several studies, nearly one-fifth of Indians with diabetes also have DR [27].

Screening: A Holistic Approach

Screening identifies undiagnosed illnesses or disorders through testing in healthy and asymptomatic persons. The World Health Organization's (WHO) requirements for a screening program are met through DR testing [28]. The condition must meet all of the following criteria: it must be a current, significant public health concern; it must have an early or latent stage that can be identified; the screening protocol must be readily accepted by both the public and healthcare professionals; it must have adequate, widely accepted treatment options; and the cost of early diagnosis and management must be taken into account concerning overall healthcare spending as well as the consequences of leaving the disease untreated [29].

New imaging modalities and biomarkers: Recent years have seen the development of several new imaging modalities, including OCT angiography (OCTA) and imaging of the retina in an ultra-wide field (UWF). UWF retinal imaging has a field of vision of about 110° to 220° and allows for visibility at least as far as the anterior margin of the ampulla of the vortex veins. Both UWF fluorescein angiography (UWFFA) and UWF color or pseudocolor photography (UWFCP) can be performed on these platforms [13]. The key benefit of UWF imaging platforms over traditional color fundus photography (CFP) is the capacity to analyze the retinal boundaries and generally have a considerably higher retinal surface area. Pupillary mydriasis is generally unnecessary while using UWF imaging systems because they are noncontact [30].

The same category and procedure may include both conventional and innovative risk factors. HbA1c, a gauge of glycemic control, is one traditional risk factor that substantially correlates with DR. Glycemic variability and other tests, like determining the plasma level of 1'5 anhydroglucitol (a dietary monosaccharide eliminated in the urine), are other components of glucose regulation [31]. Although they could be viewed as biomarkers, these factors have not been well investigated with DR. As a result, they are thought of as new biomarkers. Depending on the level of etiological disclosure, the discovery of novel biomarkers involves a process of refinement [32].

Artificial intelligence (AI): The study of theories, procedures, instruments, and software-based application systems that duplicate, magnify, and grow machine intelligence is known as AI, a vast field of computer science [33]. A branch of AI called machine learning (ML) uses statistical methods to create intelligent systems. The AI system may automatically learn and enhance its performance, such as accuracy, without being intentionally built, using either a supervised or an unsupervised approach. Deep learning (DL), which employs sophisticated machine learning (ML) algorithms, has achieved notable success in applications for computer vision and natural language [34].

AI is a useful and a crucial auxiliary tool in the early diagnosis of illnesses, especially in clinical settings with few resources. Based on fundus images and optical coherence tomography (OCT), DL has been utilized in the area of ophthalmology to treat cataracts, age-related macular degeneration (ARMD), glaucoma, DR, and glaucoma [24]. There is a lot of potential in the auxiliary diagnosis of refractive error, retinopathy of prematurity, retinal detachment, choroidal illness, and ocular malignancies. Early diagnosis is vital to avoid treatment delays and loss of sight [35].

Significance of early detection of DR

Early screening and detection of DR is crucial for several reasons [36-38]:

Early Detection

DR is a progressive eye disease that affects people with diabetes. It typically has no noticeable symptoms in the early stages, making regular screenings essential. Medical professionals can promptly initiate appropriate treatment and management strategies by detecting the condition early.

Vision Preservation

DR can lead to significant vision loss or even blindness if left untreated. However, with early detection, intervention, and proper management, the progression of the disease can be slowed down, and vision loss can be prevented or minimized. Timely treatment options such as laser therapy or medication can help preserve vision and improve outcomes.

Disease Monitoring

Regular screenings allow healthcare providers to monitor the progression of DR. By comparing the results of subsequent screenings, they can assess the effectiveness of treatment and adjust management strategies as necessary. Monitoring also helps identify any complications or additional eye conditions that may arise due to diabetes.

Treatment Options

DR has various treatment options, depending on the stage and severity of the condition. Early screening enables healthcare professionals to identify the appropriate treatment options for each patient. Treatment may include laser therapy, medication injections into the eye, or surgical intervention in advanced cases.

Comprehensive Diabetes Management

Early screening for DR is integral to comprehensive diabetes management. By addressing the ocular complications of diabetes, healthcare providers can work with patients to develop personalized treatment plans and lifestyle modifications to control blood sugar levels effectively. This holistic approach helps reduce the risk and severity of DR and other diabetic complications.

It's important to note that the frequency of screenings may vary depending on the individual's diabetes type, duration, and overall eye health. It is best to consult with a healthcare professional who can provide personalized guidance on when and how often to undergo screenings for DR [29].

Challenges in the community for DR screening and our recommendations to tackle them

Opportunistic screening is advised by the National Programme for Control of Blindness (NPCB) for the early identification of DR. To facilitate early diagnosis and suitable referral, it is important to screen persons with diabetes, including those with and without DR, at every opportunity of contact. Numerous opportunistic screening programs are already being carried out in our nation by nongovernmental organizations (NGOs), private diabetic care providers, or eye care facilities [39]. A diverse country like India will make it impossible to deploy a single DR screening strategy due to the enormous number of persons with diabetes who must be repeatedly screened [10]. However, community screening for DR faces several challenges, which can hinder early detection and intervention. Table 2 describes some common challenges faced in the community regarding DR screening and our recommendations to tackle them:

**Table 2:** Challenges in DR screening and our recommendations to deal with them DR: Diabetic retinopathy. Developed from articles [40-44].

Sl. No.	Area of Focus	Challenges	Recommendation
1.	Awareness and education	Lack of awareness among individuals with diabetes about the importance of regular eye screening for DR is a significant challenge [40].	Many people may not understand the risks and consequences of untreated DR. Thus, educating the community about the need for early detection and treatment is crucial.
2.	Access to healthcare services	Limited access to healthcare services, especially in underserved communities, can hinder screening [41].	People may need more convenient access to eye care specialists or ophthalmologists who can perform the necessary screening tests. This issue is particularly prevalent in rural areas or economically disadvantaged communities. Thus, we must increase our healthcare services to provide quality healthcare services in all parts of the country.
3.	Cost and affordability	Financial constraints can pose a significant barrier to community screening. The cost of eye examinations and diagnostic tests, such as retinal imaging, can be prohibitive for individuals without insurance or limited financial resources. The lack of insurance coverage for preventive eye care further exacerbates this challenge [41].	A targeted screening and laser treatment service for DR can be cost-effective by the standards set by the WHO. The central governments should run nationwide insurance programs to ensure even a poor person can afford quality health services. This will help reduce the disease-adjusted life years (DALY) of a person and increase the quality-adjusted life years (QALY) in their life, based on the effectiveness of the intervention. Thus, reducing the disease burden in a population and keeping the population healthy and working helps the country's economic prosperity.
4.	Health literacy and language barriers	Low health literacy and language barriers can impede effective communication and understanding of screening procedures. Individuals may need help comprehending the importance of regular eye examinations or the instructions for preparing for the screening process [42].	Health literacy can now be spread in numerous ways, including social media and television. Providing educational materials in multiple languages and ensuring clear communication can help address this challenge.
5.	Negligence-related factors	Negligence toward developing eye diseases and progressive vision loss can impact community screening efforts. Some individuals may view eye screenings as unnecessary or associate them with weakness or ageing [43].	Addressing old beliefs and promoting a positive narrative around eye health, along with emphasizing the importance of regular eye checkup, can help overcome these challenges.
6.	Compliance and follow-up	Encouraging individuals to attend screenings and follow-up appointments is crucial. However, many people may need more motivation or help prioritizing their eye health due to other competing demands or lack of symptoms [43].	Implementing reminder systems, community outreach programs, and partnerships with primary care physicians can help improve compliance and follow-up rates.
7.	Infrastructure and resources	Inadequate infrastructure and limited resources can hinder the implementation of community screening programs. This includes a need for more appropriate facilities, a shortage of qualified healthcare professionals, and limited availability of screening equipment [44].	Investment in infrastructure and allocation of adequate resources are essential to overcome these challenges.

Addressing these challenges requires a multifaceted approach involving community engagement, healthcare system improvements, targeted education campaigns, and policy changes. Collaborations between healthcare providers, community organizations, and policymakers can help develop sustainable solutions for an effective early diagnosis of the disease in the population.

## Conclusions

In conclusion, improvements in DR screening techniques have greatly aided in the early detection and preservation of vision in diabetics. The commonest cause of blindness is DR, and effective management and therapy depend on early detection. Different screening methods have been created over time, and utilizing cutting-edge technologies and strategies, which produce finely detailed and high-resolution retinal images, is one of the major developments. With the use of these imaging techniques, DR screening has become more accurate and efficient, allowing for the early identification of retinal abnormalities and rapid care.

Additionally, the submission of work that integrated the use of machine learning and computational intelligence algorithms has revolutionized DR screening. AI-based systems can precisely analyze retinal images, enabling reliable identification and classification of DR lesions. This has led to improved diagnostic accuracy and reduced dependence on ophthalmologists, making screening more accessible in resource-limited settings. The improvements in screening techniques have improved early detection and have also been crucial for maintaining vision. Early detection of DR enables surgical, intravitreal injection, and laser photocoagulation therapies to slow the course of the condition and avoid vision loss. These screening techniques have also made it easier to apply preventive interventions and improve diabetes management, which helps to preserve visual function.
